# Psychological distress among university students during remote learning in Brazil: a multicenter online study

**DOI:** 10.1590/1516-3180.2024.0299.R1.07032025

**Published:** 2025-08-29

**Authors:** Bruna Carolina Rafael Barbosa, Waléria de Paula, Aline Dayrell Ferreira Sales, Eulilian Dias de Freitas, Carolina Martins dos Santos Chagas, Helian Nunes de Oliveira, Lívia Garcia Ferreira, Luciana Saraiva da Silva, Fernanda de Carvalho Vidigal, Luciana Neri Nobre, Elaine Leandro Machado, Clareci Silva Cardoso, Adriana Lúcia Meireles

**Affiliations:** I Doctoral Student, Programa de Pós-Graduação em Saúde e Nutrição, Escola de Nutrição, Universidade Federal de Ouro Preto (UFOP), Ouro Preto (MG), Brazil.; II Pharmacist; Programa de Pós-graduação em Ciências Farmacêuticas, Escola de Farmácia, Universidade Federal de Ouro Preto (UFOP), Ouro Preto (MG), Brazil.; III Nutritionist; Professor, Departamento de Medicina Preventiva e Social, Faculdade de Medicina, Universidade Federal de Minas Gerais (UFMG), Belo Horizonte (MG), Brazil.; IV Physiotherapist; Professor, Departamento de Medicina, Universidade Federal de Juiz de Fora (UFJF), Governador Valadares (MG), Brazil.; V Nutritionist; Professor, Programa de Pós-Graduação em Nutrição e Saúde, Departamento de Nutrição, Universidade Federal de Lavras (UFLA), Lavras (MG), Brazil.; VI Psychiatrist; Professor, Departamento de Medicina Preventiva e Social, Faculdade de Medicina, Universidade Federal de Minas Gerais (UFMG), Belo Horizonte (MG), Brazil.; VII Nutritionist; Professor, Programa de Pós-Graduação em Nutrição e Saúde, Departamento de Nutrição, Universidade Federal de Lavras (UFLA), Lavras (MG), Brazil.; VIII Nutritionist; Professor, Programa de Pós-Graduação em Ciências da Saúde, Faculdade de Medicina, Universidade Federal de Uberlândia (UFU), Uberlândia (MG), Brazil.; IX Nutritionist; Professor, Programa de Pós-Graduação em Nutrição e Longevidade, Universidade Federal de Alfenas (Unifal-MG), Alfenas (MG), Brazil.; X Nutritionist; Professor, Programa de Pós-Graduação em Ciências da Nutrição, Universidade Federal dos Vales do Jequitinhonha e Mucuri (UFVJM), Diamantina (MG), Brazil.; XI Psychologist; Professor, Departamento de Medicina Preventiva e Social, Faculdade de Medicina, Universidade Federal de Minas Gerais (UFMG), Belo Horizonte (MG), Brazil.; XII Psychologist; Professor, Departamento de Medicina, Universidade Federal de São João del-Rei (UFSJ), Divinópolis (MG), Brazil.; XIII Nutritionist; Professor, Departamento de Nutrição Clínica e Social, Escola de Nutrição, Universidade Federal de Ouro Preto (UFOP), Ouro Preto (MG), Brazil.

**Keywords:** COVID-19, Mental health, Students, Multicenter study, Depression, Anxiety, Mental disorders, College students, Higher education

## Abstract

**BACKGROUND:**

Studies assessing the prevalence of mental disorders in the context of remote teaching in Brazil during the coronavirus disease (COVID-19) pandemic are scarce.

**OBJECTIVE:**

To estimate the prevalence of symptoms of anxiety and depression and their relationship with sociodemographic characteristics among university students during the COVID-19 pandemic.

**DESIGN AND SETTING:**

This multicenter cross-sectional study was conducted at eight Brazilian public universities.

**METHODS:**

This study was conducted on students regularly enrolled in undergraduate courses. Data were collected between October 2021 and February 2022 using an online self-administered questionnaire that addressed sociodemographic and academic characteristics, lifestyle habits, and health conditions. Anxiety and depression symptoms were assessed using the Depression, Anxiety, and Stress Scale-21. The variables were analyzed descriptively using frequency distribution, proportion, 95% confidence interval (95% CI), and Pearson’s chi-squared test.

**RESULTS:**

A total of 8,650 students participated, and most of them were women, white, heterosexual, cisgender, and unmarried, with a mean age of 23.9 (standard deviation: ± 6.34) years and living with family members. Symptoms of anxiety and depression were observed in 59.7% (95% CI: 58.7–60.7) and 63.0% (95% CI: 62.0–64.0) of the students, respectively. These symptoms were associated with sex, age, skin color, sexual orientation, gender identity, marital status, education of the head of the family, family income, decrease in income during the pandemic, and area of knowledge.

**CONCLUSION:**

Most university students showed symptoms of anxiety and depression during the suspension of face-to-face activities in universities, indicating the need for institutional actions and public policies aimed at promoting their health and mental well-being.

## INTRODUCTION

 Anxiety and depression are among the most prevalent mental health disorders, particularly in low-income countries. In 2015, an estimated 300 million people were reported with depression, equivalent to 4.4% of the world’s population. The estimated number of people with anxiety disorders worldwide is 264 million, totaling 3.6% of the population.^1^ The coronavirus disease (COVID-19) pandemic caused widespread concerns and raised several questions on its impacts on mental health, driven by direct psychological effects and short- and long-term social and economic consequences in the population.^2,3^

 In response to the COVID-19 pandemic, universities suspended face-to-face activities for a period and later adopted remote teaching. Studies indicate that this strategy resulted in psychological consequences among university students, possibly due to the interruption of academic routines, which compromised the progress of undergraduate courses, caused uncertainty regarding future careers, and impacted social life as interpersonal interactions were affected.^4,5^

 Moreover, prior to the pandemic, university students were identified as a group susceptible to episodes of anxiety and depression at any stage of their academic life, owing to their vulnerability to psychosocial stressors related to the challenges of their academic routine and to various changes occurring in their lives.^6,7^ The intensification of study hours and increased self-demand are factors that may worsen students’ psychological state.^8^ However, most studies that assess the prevalence of symptoms of anxiety and depression among university students are restricted to particular academic courses, such as in the health area, limiting the extrapolation of results to the university student population in general.^9-11^

 Thus, considering that university students are vulnerable to the effects of the COVID-19 pandemic, particularly with regard to mental health, the prevalence of mental disorders in this population needs to be determined during emergency remote teaching. Such information can help us understand the factors related to an increase in the prevalence of mental disorders among university students. It shall support the development and improvement of strategies and actions for structuring supportive and inclusive environments aimed at improving the physical and mental well-being of university students, considering the resumption of face-to-face academic activities. 

## OBJECTIVE

 This study aimed to estimate the prevalence of symptoms of anxiety and depression and their relationship with sociodemographic characteristics among university students from the Federal Institutions of Higher Education (IFES) in Brazil during the COVID-19 pandemic. 

## METHODS

### Study design and population

 The study "Symptoms of anxiety and depression disorder among university students in Minas Gerais: Prevalence and associated factors," also called the Project on Anxiety and Depression in University Students (PADu multicenter), is a multicenter survey conducted among undergraduate students from eight universities in the state of Minas Gerais. 

**Figure 1:**
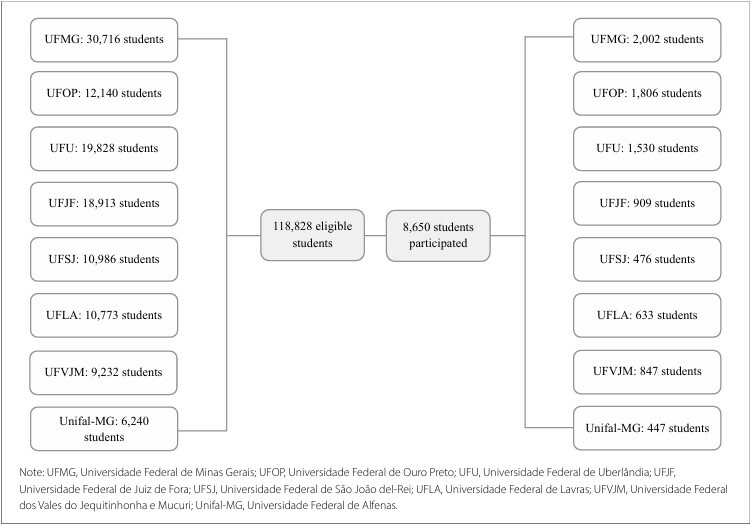
Flowchart of student participation by university. Minas Gerais, 2021–2022.

 All students who were regularly enrolled in the second half of 2021 at the eight IFES were invited to participate in the survey, and 118,828 undergraduate students were considered eligible. The PADu multicenter dataset comprised valid information from 8,650 students, with a response rate of 7.3%. Figure 1 shows a flowchart of university student participation. 

 The inclusion criteria were age ≥ 18 years, students enrolled in any undergraduate course at the eight IFES participating in the project, and those enrolled in any academic period. Students who did not complete the questionnaire, postgraduate and residency students, or those who were regularly enrolled but did not participate in academic activities or were on exchange during data collection were excluded from the survey. As the questionnaire was online and any student could access it through social networks, graduate students and residents participated in this study. Only data from undergraduate students who met the inclusion criteria were retained. 

### Data collection

 Data collection was conducted between October 2021 and February 2022, which lasted for three months at each participating IFES. The study was approved by the Research Ethics Committees of all participating universities. 

 The survey was widely publicized on the websites and social networks of the IFES and PADu (@padufederais), in addition to tutoring programs, laboratories, study and research groups, centers, and academic directories. As a communication and recruitment strategy for participants, a visual identity was developed and applied to all promotional materials, social networks, questionnaires, and materials used by the team. 

 Students received an invitation text and a link to access the self-administered confidential questionnaire via email, which was available on Google Forms. Participation in the study was initiated when accessing the questionnaire upon agreement through an online check of the Free and Informed Consent Form presented electronically and available for download. All questionnaire items were presented, detailing important points and possible areas of confusion. 

 For data collection, the study required the participation of postgraduate students (doctoral and master’s students) and scientific initiation. Each IFES has a reference researcher and at least one graduate or scientific initiation student to assist with the project. The team received prior training from the coordinating center (Universidade Federal de Ouro Preto [UFOP]) to standardize data collection at different IFES. During training, the researchers were informed of the importance of the research, topics addressed in the questionnaire, and the methodological criteria of the study, in addition to the method of sending the questionnaire link and approaching individuals for dissemination. In addition, a field manual containing information about the project, general instructions for collection, schedule of activities, and sending of e-mails, was prepared. The entire data collection process was monitored by a general research coordinator, aiming to maintain consistency in data collection procedures and homogeneity of information, and consequently to obtain reliable data and reduce the chance of possible errors and biases, such as duplicates and typographical errors. 

### Data collection instrument

 The questionnaire used in the PADu multicenter study included questions specifically designed for the study, which were prepared by a team of researchers, and questions used in national surveys. Questions related to general, sociodemographic, and academic characteristics; lifestyle habits; health conditions; social support; quality of life; and resilience were included (Table 1).^12-31^

**Table 1:** Topics included in the questionnaire of the Project on Anxiety and Depression among University Students, 2021–2022

**Modules**	**Variables**	**References**
Student identification	Enrollment,* course, and institution.	Questions prepared by the project researchers.
General characteristics and socioeconomic conditions	Age, city of residence and housing, race, biological sex, gender identity, sexual orientation, level of education of the head of the family, marital status, family income, and religious belief.	Questions taken from and/or based on the Instituto Brasileiro de Geografia e Estatística^12^ and Pesquisa Nacional de Saúde (PNS)^13^ census.
Life habits	Number of participants in remote teaching, difficulties in dealing with the pandemic, and study routine. Consumption of alcohol, tobacco, and illicit substances. Practice of physical exercise and sedentary behavior. Frequency and eating habits, self-efficacy for adopting healthy practices, emotional eating, body image issues, and dysfunctional eating attitudes.	Questions prepared by the project researchers. Questions taken from and/or based on the Instituto Brasileiro de Geografia e Estatística,^12^ PNS,^13^ and Sistema de Vigilância de Fatores de Risco e Proteção para Doenças Crônicas por Inquérito Telefônico (VIGITEL)^14^ census. Questions based on VIGITEL.^14^ Instrument constructed from items addressed in previous research with adolescents and young adults.^15-21^ The Three Dietary Factors Questionnaire was used to analyze the profile of eating behavior according to the emotional eating subscale score.^22^ The translated and validated version for the Brazilian population was used.^23^ Simplified questionnaire of dysfunctional eating attitudes based on the study by Ferreira and Veiga (2008).^24^
Health conditions	General health aspects, such as self-reported weight and height, presence of morbidities, self-rated health, suicidal ideation, and medication use.	Questions taken and/or adapted from the PNS,^13^ use of medication adapted from the study by Bertoldi et al.,^25^ and study of self-injury adapted from Fonseca et al.^26^
Anxiety, depression, and stress scale	Variables that assess symptoms of anxiety, depression, and stress.	Depression Anxiety Stress Scale-21, translated and validated by Vignola and Tucci.^27^
Social support scale	Variables that assess social support.	Social support satisfaction scale, version adapted from Ribeiro^28^ and Zanini et al.^29^
Quality of life scale	Variables that assess quality of life.	Quality of life determines World Health Organization Quality of Life abbreviated version, translated and validated by Fleck et al.^30^
Resilience scale	Variables that assess resilience.	Connor–Davidson Resilience Scale, translated and validated by Lopes and Martins.^31^

* The UFU Research Ethics Committee did not authorize the collection of this information to avoid identifying students.

 To select the investigated topics, the researchers considered the available evidence regarding the outcomes of interest, comparability with similar studies, validated instruments with open access, and meeting the research objectives of the eight IFES. 

### Study variables

 The presence of symptoms of anxiety and depression in the health condition block was assessed using the Portuguese version of the Depression Anxiety Stress Scale-21 (DASS-21), adapted and validated by Vignola and Tucci. The DASS-21 is a self-reported test composed of a set of three independent subscales, with seven questions each. The scale assesses symptoms of anxiety, depression, and stress presented by individuals in the last week, that is, the week prior to data collection, through the total scores obtained in the subscales.^27^ The subscale items are divided as follows: the depression subscale includes questions 3, 5, 10, 13, 16, 17, and 21; the anxiety subscale includes questions 2, 4, 7, 9, 15, 19, and 20; and the stress subscale includes questions 1, 6, 8, 11, 12, 14, and 18.^32^

 Responses to the items were structured on a four-point Likerttype scale ranging from 0 (did not apply at all) to 3 (applied a lot or most of the time). The answers "applied to some degree or for a short time" and "applied to a considerable degree or for a good part of the time" refer to scores 1 and 2, respectively. The scores for anxiety and depression are generated from the total scores and are then multiplied by 2, generating the classification levels as "normal," "mild," "moderate," "severe," and "extremely severe." 

 In the present study, only symptoms of anxiety and depression were evaluated. Based on the classification presented above, the "moderate," "severe," and "extremely severe" levels were recategorized as "moderate-to-extremely severe" and the "normal" and "mild" levels as "normal and mild." 

 The sociodemographic variables used to describe the sample included sex (male and female), age (18–20, 21–22, 23–25, and ≥ 26 years of age), skin color (white, brown, black, and yellow, indigenous, and others), sexual orientation (heterosexual, homosexual, bisexual, asexual, and others), gender identity (cisgender, transgender, and non-binary), marital status (single, married/stable union, widowed, and divorced), housing (with and without family members), education of the head of the family (no education or incomplete primary education, complete primary education or incomplete secondary education, complete secondary education or incomplete higher education, and complete higher education), total family income (≤ 1 to 2 minimum wages, 3 to 5 minimum wages, 6 to 10 minimum wages, and > 10 minimum wages), and decrease in family income in the three months prior to the survey (no and yes). The salary value considered in this study refers to the minimum wage in force in 2021 (R$ 1,100). 

 Academic aspects related to the area of knowledge in the course (life sciences, exact sciences, and human, social, and applied sciences) were also investigated and used to describe the data. 

### Statistical analyses

 The use of an online questionnaire enabled the development of an automatic database that was exported to Microsoft Excel 2013. Subsequently, data coding and consistency analyses were performed to ensure the quality and validity of the information. 

 For sample characterization and data comparison, variables were analyzed using frequency distribution and Pearson’s chisquared test. The proportion and 95% confidence interval (95% CI) were used to estimate the prevalence of symptoms of anxiety and depression. Statistical significance was set at 5%. Analyses were performed using the Stata statistical software (version 13.0; Stata Corporation, College Station, TX, USA). 

### Ethical aspects

 The PADu multicenter study was conducted according to the guidelines established by the Declaration of Helsinki and was approved by the Research Ethics Committee of the coordinating center (UFOP) under protocol number 43027421.3.1001.5150 and by the Ethics Committees of all institutions (Universidade Federal de Minas Gerais [UFMG]: 43027421.3.2004.5149; Universidade Federal de Uberlândia [UFU]: 43027421.3.2001.5152; Universidade Federal de Juiz de Fora [UFJF]: 43027421.3.2003.5147; Universidade Federal de São João del-Rei [UFSJ]: 43027421.3.2002.5545; Universidade Federal de Lavras [UFLA]: 43027421.3.2006.5148; Universidade Federal dos Vales do Jequitinhonha e Mucuri [UFVJM]: 43027421.3.2009.5108; and Universidade Federal de Alfenas [Unifal-MG]: 43027421.3.2008.5142). All participants were duly informed about voluntary collaboration and guaranteed anonymity, research objectives, steps to be undertaken, and risks and benefits of their participation before signing the Free and Informed Consent Term and approval by the Ethics Committees. 

## RESULTS

 Of the 8,650 participating students, the majority were women, with a mean age of 23 years and 9 months (standard deviation ± 6.34 years), white, heterosexual, cisgender, single, and living with family members. Approximately 41.0% of the students reported a family income of three to five times the minimum wage, and 51.6% reported a decrease in family income three months prior to data collection. Regarding the education of the family head, 39.4% of the students reported completing higher education. Students reported being enrolled in courses in the following areas of knowledge: 39.5% exact sciences, 31.9% life sciences, and 28.9% human, social, and applied sciences. Table 2 presents the main sociodemographic and academic characteristics of the participants. 

**Table 2:** Sociodemographic characteristics of university students according to the presence of symptoms of anxiety and depression during the COVID-19 pandemic. Project on Anxiety and Depression in University Students, 2021–2022 (n= 8,650)

**Variables**		**Total (n, %)**	**Anxiety symptoms**		P value*	**Depression symptoms**		P value*
			**Normal and mild (n = 3,486)z**	**Moderate to extremely severe (n = 5,164)**		**Normal and mild (n = 3,201)**	**Moderate to extremely severe (n = 5,449)**	
**Biological sex (n = 8,615)**								
	*Male*	2,955 (34.3)	1,560 (52.8)	1,395 (47.2)	**< 0.001**	1,234 (41.8)	1,234 (41.8)	**< 0.001**
	*Female*	566 (65.7)	1,913 (33.8)	3,747 (66.2)		1,959 (34.6)	1,959 (34.6)	
**Age**								
	*18–20 years*	2,552 (29.5)	969 (38)	1,583 (62)	**< 0.001**	977 (38.3)	1,575 (61.7)	**0.006**
	*21–22 years*	2,135 (24.7)	855 (40)	1,280 (60)		789 (37)	1,346 (63)	
	*23–25 years*	2,000 (23.1)	759 (38)	1,241 (62)		677 (33.9)	1,323 (66.1)	
	*≥ 26 years*	1,963 (22.7)	903 (46)	1,060 (54)		758 (38.6)	1,205 (61.4)	
**Skin color (n = 8,474)**								
	*White*	4,694 (55.4)	1,953 (41.6)	2,741 (58.4)	**0.016**	1,833 (39)	2,861 (61)	**< 0.001**
	*Brown*	2,622 (30.9)	1,047 (39.9)	1,575 (60.1)		961 (36.7)	1,661 (63.3)	
	*Black*	1,039 (12.3)	382 (36.8)	657 (63.2)		307 (29.6)	732 (70.4)	
	*Yellow, Indigenous, and others*	119 (1.4)	55 (46.2)	64 (53.8)		45 (37.8)	74 (62.2)	
**Sexual orientation (n = 8,374)**								
	*Heterosexual*	5,714 (68.2)	2,614 (45.8)	3,100 (54.2)	**< 0.001**	2,412 (42.2)	3,302 (57.8)	**< 0.001**
	*Homosexual*	753 (9)	254 (33.7)	499 (66.3)		217 (28.8)	536 (71.2)	
	*Bisexual*	1,685 (20.1)	467 (27.7)	1,218 (72.3)		448 (26.6)	1,237 (73.4)	
	*Asexual and others*	222 (2.7)	51 (23)	171 (77)		37 (16.7)	185 (83.3)	
**Gender identity (n = 8,433)**								
	*Cisgender*	8,211 (97.4)	3,353 (40.8)	4,858 (59.2)	**< 0.001**	3,075 (37.4)	5,136 (62.6)	**< 0.001**
	*Transgender*	59 (0.7)	17 (28.8)	42 (71.2)		24 (40.7)	35 (59.3)	
	*Non-binary*	163 (1.9)	42 (25.8)	121 (74.2)		30 (18.4)	133 (81.6)	
**Marital status**								
	*Single*	7,775 (90.6)	3,067 (39.4)	4,708 (60.6)	**< 0.001**	2,819 (36.3)	4,956 (63.7)	**< 0.001**
	*Married/stable union*	709 (8.3)	356 (50.2)	353 (49.8)		321 (45.3)	388 (54.7)	
	*Widowed and divorced*	98 (1.1)	42 (42.9)	56 (57.1)		44 (44.9)	54 (55.1)	
**Housing**								
	*Without family members*	2,048 (23.7)	2,697 (40.9)	3,905 (59.1)	0.061	2,467 (37.4)	4,135 (62.6)	0.211
	*With family members*	6,602 (76.3)	789 (38.5)	1,259 (61.5)		734 (35.8)	1,314 (64.2)	
**Education of the head of the family (n = 8,528)**								
	*No education or incomplete primary education*	1,297 (15.2)	429 (33.1)	868 (66.9)	**< 0.001**	405 (31.2)	892 (68.8)	**< 0.001**
	*Complete primary education or incomplete secondary education*	941 (11)	371 (39.4)	570 (60.6)		342 (36.3)	599 (63.7)	
	*Complete secondary education or incomplete higher education*	2,931 (34.4)	1,171 (40)	1,760 (60)		1,035 (35.3)	1,896 (64.7)	
	*Complete higher education*	3,359 (39.4)	1,463 (43.6)	1,896 (56.4)		1,381 (41.1)	1,978 (58.9)	
Total family income**								
	*≤ 1–2 minimum wages*	2,595 (32.1)	812 (31.3)	1,783 (68.7)	**< 0.001**	754 (29.1)	1,841 (70.9)	**< 0.001**
	*3–5 minimum wages*	3310 (40.9)	1,383 (41.8)	1,927 (58.2)		1,219 (36.8)	2,091 (63.2)	
	*6–10 minimum wages*	1,397 (17.3)	642 (46)	755 (54)		603 (43.2)	794 (56.8)	
	*> 10 minimum wages*	788 (9.7)	416 (52.8)	372 (47.2)		404 (51.3)	384 (48.7)	
**Drop in family income (n = 8,195)**								
	*No*	3,966 (48.4)	1,854 (46.8)	2,112 (53.2)	**< 0.001**	1,704 (43)	2,262 (57)	**< 0.001**
	*Yes*	4,229 (51.6)	1,463 (34.6)	2,766 (65.4)		1,332 (31.5)	2,897 (68.5)	
**Area of knowledge**								
	*Exact Sciences*	3,416 (39.5)	1,494 (43.7)	1,922 (56.3)	**< 0.001**	1,310 (38.3)	2,106 (61.7)	**< 0.001**
	*Life Sciences*	2,731 (31.6)	1,151 (42.1)	1,580 (57.9)		1,092 (40)	1,639 (60)	
	*Humanities and Social and Applied Sciences*	2,503 (28.9)	841 (33.6)	1,662 (66.4)		799 (31.9)	1,704 (68.1)	

Note: Variables with n less than 8,650 differ from the total study sample because of missing responses.

* P value obtained by chi-squared test; values in bold indicate statistically significant variables.

** Minimum wage in force in Brazil in 2021 = R$ 1,100.

 Univariate analysis (Table 2) showed that sex, age, skin color, sexual orientation, gender identity, marital status, education of the family head, family income, decrease in family income, and area of knowledge were related to symptoms of anxiety and depression among the students (P < 0.050). 

**Figure 2:**
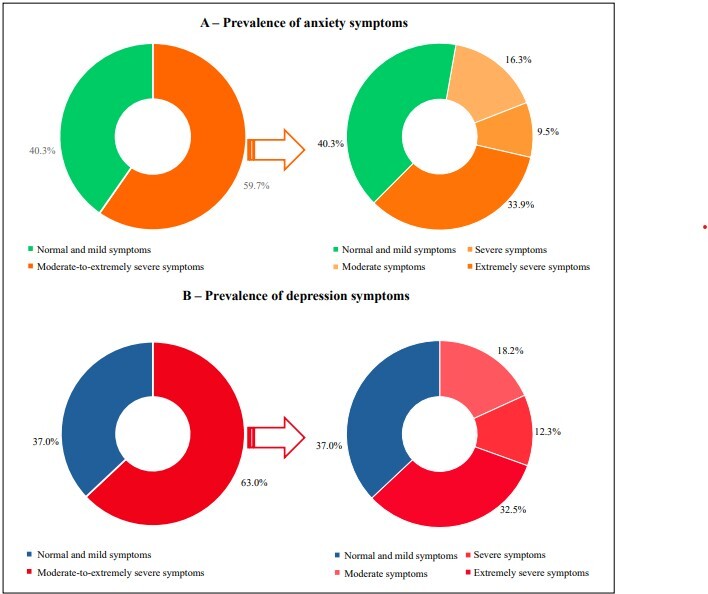
Prevalence of symptoms of anxiety and depression in university students. Project on Anxiety and Depression in University Students, 2021–2022 (n = 8,650).

 Among the participants, 59.7% (95% CI: 58.7–60.7) were classified as having anxiety symptoms; of them, 33.9% had extremely severe symptoms. The prevalence of symptoms of depression among students was 63.0% (95% CI: 62.0–64.0); of them, 32.5% were classified as having extremely severe symptoms (Figure 2). 

**Figure 3:**
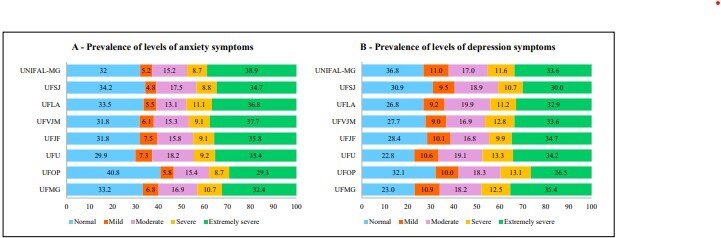
Prevalence of symptoms of anxiety and depression in university students, according to participating federal institutions of higher education. Project on Anxiety and Depression in University Students, 2021–2022 (n = 8,650).

Figure 3 shows the prevalence of symptoms of anxiety and depression according to their severity. Students from the UFOP had a higher proportion of absence of symptoms of anxiety (40.8%) and depression (32.1%), considering the normal classification, and a lower proportion of extremely severe symptoms of both anxiety (29.3%) and depression (26.5%). When compared to the IFES, a higher prevalence of extremely severe anxiety symptoms was observed among students from Unifal-MG (38.9%) and UFVJM (37.7%). However, a higher proportion of students with symptoms of extremely severe depression was observed in the UFMG (35.4%) and UFJF (35.7%). 

## DISCUSSION

 The results of this study indicated a high prevalence of symptoms of mental disorders among students investigated at the eight IFES in Minas Gerais during the COVID-19 pandemic. In addition, a significant difference was observed in the prevalence of moderate-to-extremely severe symptoms of anxiety and depression in relation to sociodemographic and academic characteristics. 

 During the COVID-19 pandemic, high prevalence rates of mental disorders have been estimated in the world population. Different meta-analyses indicate prevalences between 26.9–38.1% and 28.0–34.3% for symptoms of anxiety and depression in the general population, respectively.^33-36^ In the pandemic context, Santomauro et al.^3^ indicated an increase of 25.6% in anxiety diagnoses and 27.6% in new cases of depression. In a household epidemiological survey conducted on adults over 18 years of age during the pandemic between October and December 2020 in two cities in the state of Minas Gerais, the presence of anxiety symptoms was observed in 23.3% of participants and depression symptoms in 15.6%.^37^

 In the university population, the prevalence of anxiety and depression during the pandemic was similar to or higher than that in the general population,^38,39^ corroborating the findings of the present study. The results indicate an increase in the prevalence of mental disorders among university students during the COVID-19 pandemic when compared to studies conducted before this period.^40,41^ In a study conducted in 2019 on students in the first semester at a public university, which used the DASS-21 scale, De Paula et al.^40^ observed that the self-reported prevalence of symptoms of anxiety and depression among university students was 42.5% (95% CI: 37.4–47.7) and 33.2% (95% CI: 28.3–38.2), respectively, suggesting lower levels of mental disorders than those found in the present study during the pandemic. 

 In a survey of students at a university in the United States in the first half of 2020, Wang et al.^42^ found that 48.1% of undergraduate and graduate students had moderate-to-severe depression, whereas 38.5% of students had mild-to-severe anxiety. The authors also found that most participants (71.3%) reported increased stress and anxiety levels during the COVID-19 pandemic owing to the abrupt transition and maintenance of online classes, concerns about grades, and late graduation. The prevalences found by Wang et al.^42^ are lower than those found in the present study; however, they showed an increased prevalence of mental disorders among university students during the pandemic. 

 Thus, the physical closure of educational institutions and the suspension of face-to-face activities during the COVID-19 pandemic resulted in challenges for the academic community and society^43^ with psychological implications for university students.^5^ The period of distance teaching and learning was unprecedented and could have resulted in psychological consequences among university students.^4,43^ The higher prevalence of symptoms of anxiety and depression in this population could be a consequence of the drastic change in the routine of the university community, with significant effects in the short and long terms. 

 In the present study, the estimates of the prevalence of symptoms of anxiety and depression varied according to the sociodemographic and academic characteristics of university students, such as sex, age, skin color, sexual orientation, gender identity, marital status, education of the family head, family income, decreased income during the pandemic, and area of knowledge. Evidence shows that mental disorders are more prevalent among female students, those who are older and of low socioeconomic status, those among ethnic, racial, and sexual minorities (homosexual and bisexual), and students in health courses.^ 40,41, 44,45^ For example, gender differences may be correlated with personal stigma, vulnerability, prejudice, and gender discrimination, in addition to the fact that non-heterosexual women recognize symptoms of depression better and seek more help from counseling and health services than men and heterosexual women.^41,45^

 The findings of this study are consistent with those of other studies conducted in the context of the pandemic, demonstrating that culturally disadvantaged and vulnerable groups, such as women, those of black origin and minority ethnicity, those with lower socioeconomic status, and those among sexual minorities, have a higher prevalence of symptoms of anxiety and/or depression.^2,46^ Studies conducted on students in France between April and May 2020 observed that being of a female or nonbinary gender, reporting a decrease in income, poor-quality housing, and not living with family members were risk factors associated with mental health problems among students who experienced the COVID-19 pandemic.^46^

 This scenario may be understandable, considering the magnitude of changes in academic routines owing to the inclusion of remote teaching.^47^ However, universities play a fundamental role in supporting university students and in the development of public policies that focus on promoting mental health, as well as actions and strategies to better address the effects of the pandemic. In addition, considering that universities represent an opportune environment for health promotion, institutions should contribute to supporting networks and welcome counseling centers for students to share coping resources and encourage them to take measures to protect their mental health.^7^

 The findings of this study have some limitations. First, the inherent limitation of the adopted design, owing to its cross-sectional nature, did not allow for an assessment of the impact of the pandemic on the occurrence of mental disorders. Another limitation is the non-probabilistic sample in which all students were invited to participate. Thus, the possibility of response bias was highlighted. Those with a previous diagnosis of a mental disorder or related difficulties were possibly more likely to participate in the study, which may have contributed to the overestimation of the prevalence of symptoms of mental disorders found in the present study. In addition, the presence of symptoms of anxiety and depression was measured using a self-reported scale that assesses the symptoms and is not based on a medical diagnosis of the disease. The stratification of the classifications adopted in this study may differ from those adopted by other authors in national and international studies. Furthermore, different measurement tools and cut-off points have been used to assess the presence of symptoms of anxiety and depression, which may have influenced the comparison of the studies presented here. Although the results indicated a high prevalence of mental disorders among students, interpretation and comparison with other studies should be conducted with caution 

 Despite these limitations, this study has strengths and expands the scientific knowledge about mental disorders among university students during the COVID-19 pandemic. It was conducted with a large sample of university students from eight public institutions of higher education, and the findings presented must be considered at both the state and local levels so that actions for the protection, control, and reduction of mental disorders can be targets for sustainable and effective policies in the university population. 

## CONCLUSION

 The results showed a high prevalence of symptoms of anxiety and depression among students during the suspension of face-to-face activities in universities. Thus, together with other findings in the literature, this study adds relevant evidence to guide more cost-effective actions toward priority groups, in addition to providing evidence of possible damages resulting from the pandemic that require attention, as they may have short- and long-term consequences. Furthermore, considering that universities represent an opportune environment for promoting health, institutions must create actions such as psychological counseling services and support groups as well as develop policies that support diversity and inclusion aimed at the physical and mental well-being of students. 
